# Interleukin-10-Producing DC-10 Is a Unique Tool to Promote Tolerance *Via* Antigen-Specific T Regulatory Type 1 Cells

**DOI:** 10.3389/fimmu.2018.00682

**Published:** 2018-04-06

**Authors:** Michela Comi, Giada Amodio, Silvia Gregori

**Affiliations:** San Raffaele Telethon Institute for Gene Therapy (SR-Tiget) San Raffaele Scientific Institute IRCCS, Milan, Italy

**Keywords:** interleukin-10, dendritic cells, tolerance, DC-10, T regulatory type 1 cells

## Abstract

The prominent role of tolerogenic dendritic cells (tolDCs) in promoting immune tolerance and the development of efficient methods to generate clinical grade products allow the application of tolDCs as cell-based approach to dampen antigen (Ag)-specific T cell responses in autoimmunity and transplantation. Interleukin (IL)-10 potently modulates the differentiation and functions of myeloid cells. Our group contributed to the identification of IL-10 as key factor in inducing a subset of human tolDCs, named dendritic cell (DC)-10, endowed with the ability to spontaneously release IL-10 and induce Ag-specific T regulatory type 1 (Tr1) cells. We will provide an overview on the role of IL-10 in modulating myeloid cells and in promoting DC-10. Moreover, we will discuss the clinical application of DC-10 as inducers of Ag-specific Tr1 cells for tailoring Tr1-based cell therapy, and as cell product for promoting and restoring tolerance in T-cell-mediated diseases.

## Introduction

Interleukin (IL)-10 is a powerful anti-inflammatory cytokine that plays an essential role in dampening immune responses and in preventing chronic inflammatory pathologies (1). Deficiency or aberrant expression of IL-10 or IL-10 receptor (IL-10R) enhance inflammatory responses to microbial challenge and lead to the development of inflammatory bowel disease (2–4) and several autoimmune diseases [as reviewed in Ref. (5, 6)]. Some pathogens can harness the immunosuppressive capacity of IL-10 to limit host immune responses, leading to persistent infection [as reviewed in Ref. (7)].

Human IL-10 was cloned (8) from a tetanus toxin-specific CD4^+^ human T-cell clone isolated from peripheral blood of a patient with severe combined immunodeficiency successfully transplanted with fetal liver and thymus, who spontaneously developed tolerance (9). From its discovery, IL-10 has been demonstrated to be produced by almost all leukocytes, including all T cell subsets, monocytes, macrophages, dendritic cells (DCs), B and natural killer (NK) cells, mast cells, neutrophils, and eosinophils [reviewed in Ref. (10)]. In addition, epithelial cells and keratinocytes can also secrete IL-10 in response to infection or tissue damage as well as tumor cells (11, 12).

Interleukin-10 upon interaction with IL-10R regulates the expression of several genes resulting in the downregulation of pro-inflammatory mediators, the inhibition of antigen (Ag) presentation, and the upregulation of immune-modulatory molecules. Overall, IL-10 modulates antigen-presenting cells (APCs), inhibits, directly and indirectly, effector T cell proliferation and cytokine production, and promotes regulatory cell differentiation [reviewed in Ref. (13, 14)].

Here, we present an overview on the role of IL-10 in promoting the differentiation of myeloid regulatory DCs, focusing on the induction of a subset of human tolerogenic (tol) DCs, termed DC-10. Moreover, we discuss the role of DC-10 in modulating T cell responses *in vitro* and *in vivo* and the current clinical application of DC-10 for cell-based therapeutic approaches.

## IL-10 and Modulation of Myeloid Cells

**Figure 1:**
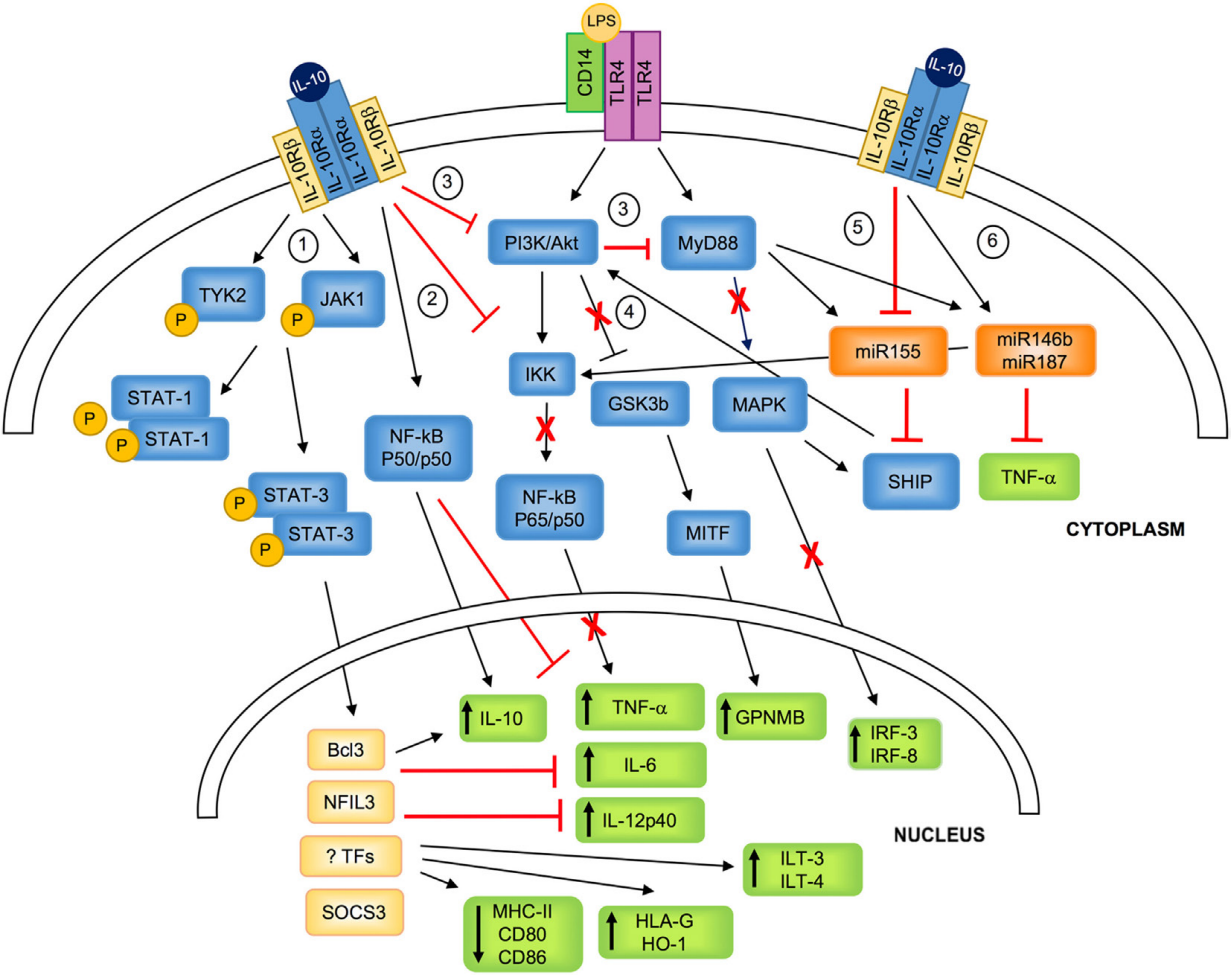
IL-10-mediated modulation of myeloid cells. IL-10 binds to a tetrameric receptor consisting of two IL-10Rα and two IL-10Rβ subunits. 1. IL-10/IL-10R interaction leads to JAK1 and TYK2 phosphorylation and the consequent STAT3 and STAT1 phosphorylation. P-STATs, and in particular P-STAT3, dimerizes and translocates to the nucleus, where it promotes the transcription of specific molecules (i.e., SOCS3) or transcription factors (i.e., BCL3 and NFIL3), and inhibits the transport of MHC class II to the plasma membrane. 2. IL-10 signaling inhibits LPS-mediated activation of IKK that in turn prevents NF-kB-p65/p50 nuclear translocation and the expression of pro-inflammatory cytokine. In parallel, IL-10 promotes the selective NF-kB-p50/p50 nuclear translocation, which concurs in downregulating pro-inflammatory cytokine expression, and, in association with BCL3, promotes IL-10 expression. 3. IL-10 inhibits PI3K/Akt pathway that prevents LPS-mediated activation of MyD88, resulting in the inhibition of the expression of IRF-3 and IRF-8. 4. IL-10-mediated inhibition of PI3K/Akt pathway leads to GSK3β and MITF activation, responsible for the upregulation of the transcription of GPNMB. 5. IL-10 downmodulates LPS-induced expression of miR155, which directly inhibits SHIP1 and favors the negative regulation of TLR4 signaling by counteracting PI3K activity. 6. IL-10 enhances LPS-mediated induction of miR146b and miR187, which post-transcriptionally regulate mRNA encoding for TNF-α and reduce IL-6 and IL-12p40 transcription *via* inhibition of the transcription factor IkB. TYK, tyrosine kinase; JAK, Janus kinase; PI3K, phosphoinositide 3-kinase; Akt, protein kinase B; MyD88, myeloid differentiation primary response 88; STAT, signal transducer and activator of transcription; IKK, IkB kinase; NF-kB, nuclear factor kappa-light-chain-enhancer of activated B cells; GSK3β, glycogen synthase kinase 3 beta; MITF, microphthalmia-associated transcription factor; MAPK, mitogen-activated protein kinase; SHIP1, SH-2 containing inositol 5′ polyphosphatase 1; Bcl3, B-cell lymphoma 3-encoded protein; NFIL3, nuclear factor interleukin 3; TFs, unknown transcription factors; SOCS, suppressor of cytokine signaling; GPNMB, glycoprotein NMB; HO-1, heme-oxygenase-1; ILT, immunoglobulin-like transcript; IRF, interferon regulatory factor; IL-10R, IL-10 receptor.

Interleukin-10 signaling in monocytes/macrophages and DCs converges, *via* several mechanisms, to regulate nuclear transcriptional events, inducing the initiation of homeostatic and anti-inflammatory programs. IL-10 interacts with a tetrameric receptor consisting of two IL-10Rα and two IL-10Rβ subunits. IL-10Rα binds IL-10, while IL-10Rβ, interacting with accessory molecules, mediates intracytoplasmic signals (14). IL-10/IL-10R interaction leads to phosphorylation of Janus kinase 1 (JAK1) associated with IL-10Rα and of Tyrosine Kinase 2 (TYK2), associated with IL-10Rβ. These kinases further phosphorylate two tyrosine residues located on the intracellular domain of IL-10Rα that act as temporary docking sites for STAT3 and STAT1 (15). Phosphorylated STATs homo/hetero-dimerize and translocate into the nucleus, where they bind to STAT-responsive genes (1, 16). Although the mechanisms underlying the IL-10/STAT3-mediated responses are still to be fully understood, it has become evident that both IL-10 and STAT3 are required for anti-inflammatory responses. In macrophages, one of the major effects of IL-10/STAT3-mediated signaling is the transcription inhibition of up to 20% of the LPS-induced genes (17). This anti-inflammatory activity is mediated primarily by STAT3 that, upon nuclear translocation, promotes the expression of specific genes, including those encoding for transcription factors, the ultimate effectors of the IL-10-mediated anti-inflammatory responses (18). Among molecules involved in inhibiting activation of myeloid cells, BCL3 has been shown to suppress LPS-induced TNF-α expression by inhibiting NF-kB (19), and NFIL3 has been demonstrated to specifically target a distal enhancer of *Il12b* and repress IL-12p40 expression (20, 21). IL-10/STAT3-mediated signal in macrophages promotes the expression of suppressor of cytokine signaling 3 (SOCS3) (22), a member of the SOCS protein family that plays important roles in the negative regulation of cytokine signaling pathways (23) (Figure 1). Although both IL-10 and IL-6 promote *via* STAT3 the expression of SOCS3, its inhibitory effects are restricted to IL-6R-mediated signaling (16). This evidence indicates that SOCS3 plays a role in regulating the pro-inflammatory effects of IL-6 (24).

In macrophages, upon activation with LPS or TNF-α, IL-10 prevents the activation and nuclear translocation of the classical NF-kB by inhibiting IkB kinase (IKK) activity (25–27), and hampers NF-kB DNA binding (28). This mechanism has been applied also to *in vitro* differentiated myeloid DCs, in which pre-treatment with IL-10 results in NF-kB inhibition that correlates with suppression of IKK and Akt activities (29). Similarly, the addition of IL-10 during TLR-mediated activation of monocyte-derived DCs hinders MyD88 signaling, leading to the downregulation of NF-kB family members c-Rel and p65, and interferon regulatory factor (IRF)-3 and IRF-8, an effect mediated by the inhibitory activity on the PI3K/Akt pathway (30). The IL-10-mediated inhibition of the PI3K/Akt signaling pathway leads also to the activation of the glycogen synthase kinase 3 beta (GSK3beta) and of the downstream microphthalmia-associated transcription factor (MITF) that translocates to the nucleus and drives the expression of the inhibitory molecule glycoprotein (GP) NMB (30) (Figure 1). At steady state and upon activation of myeloid cells, IL-10 signaling induces the selective nuclear translocation of NF-kB p50/p50, overall preventing the expression of several pro-inflammatory mediators, including IL-6 and MIP-2α (27). Interestingly, in activated macrophages, BCL3, a member of the IkB protein family localized in the nucleus and tightly associated with NF-kB p50 (31), acts to repress the transcription of pro-inflammatory cytokines, and positively regulates the expression of IL-10 (32).

An additional effect of IL-10 in myeloid cells is the downregulation of MHC class II (33, 34) and costimulatory molecules (35) expression (Figure 1). The mechanism of IL-10-mediated deregulation of MHC class II expression involves the transport inhibition of mature and peptide-loaded MHC class II complex to the plasma membrane (36). These IL-10-mediated effects are completely reversed by blocking STAT3 (37), although the role of STAT3 in these mechanisms has not been fully elucidated.

Interleukin-10 regulates at post-transcriptional levels, *via* micro (mi)RNAs, the expression of pro-inflammatory cytokines (38). IL-10 inhibits the expression of LPS-induced miR155, allowing the expression of SH-2 containing inositol 5′ polyphosphatase 1 (SHIP-1), which in turn negatively regulates PI3K-mediated activation of NF-kB and MAPK, and switches off the pro-inflammatory response (39). On the contrary, upon LPS stimulation, IL-10 rapidly and transiently enhances miR146b and sustains miR187 expression in myeloid cells. miR187 acts as negative modulator of LPS responses by directly limiting TNF-α production at post-transcriptional level and by reducing IL-6 and IL-12p40 transcription *via* silencing the transcription factor IkB (40) (Figure 1). miRNAs have been also involved in regulating IL-10 expression upon LPS-mediated activation: upregulation of miR21 indirectly increases IL-10 production *via* downregulation of programmed cell death 4 (41). Overall, these evidences indicate that, through a complex network of miRNAs, IL-10 drives anti-inflammatory responses by upregulating miR146b and miR187 and by downregulating pro-inflammatory miRNAs, such as miR155.

In summary, IL-10 directly and indirectly, *via* inducing STAT3 responsive genes and/or modulating NF-kB and MAPK activities, inhibits pro-inflammatory cytokine gene transcription in activated myeloid cells, and the expression of MHC class II and costimulatory molecules, overall preventing the ability of myeloid cells to efficiently present Ags to T cells and to activate effector T cells [reviewed in Ref. (7)].

Besides being an anti-inflammatory mediator, IL-10 promotes the expression of several tolerogenic molecules in human monocytes, macrophages, and DCs, including IL-10 itself (15), heme-oxygenase (HO-1) (42, 43), and immunoglobulin-like transcript 3 (ILT3) and ILT4 (44). HO-1 is a protein of heme degradation pathway playing a central role in tissue homeostasis and protection against oxidative stress (42). HO-1 is involved in the polarization of anti-inflammatory macrophages, which in turn acquire the ability to secrete high levels of HO-1 (45, 46). In human DCs, HO-1 inhibits their ability to stimulate allogeneic (allo) T cells and promotes their suppressive effects (43). ILT3 and ILT4 display a long cytoplasmic tail containing immune-receptor tyrosine-based inhibitory motifs that upon binding to HLA class I molecules transduce a negative signal through the recruitment of the tyrosine phosphatase SHP-1. This leads to inhibition of NF-kB activation and, consequently transcription of genes encoding for costimulatory molecules (47, 48). Finally, IL-10 upregulates the transcription of the non-classical HLA class I molecule HLA-G (49, 50), one of the ILT4 ligands (47) with known immune-modulatory functions.

Overall, IL-10 *via* several mechanisms regulates activation and function of myeloid cells, thereby playing an important role in modulating immune responses in healthy and pathological conditions.

## IL-10-Mediated Modulation of Monocyte-Derived DCs

Interleukin-10 has been repetitively applied to modulate *in vitro* differentiation of monocyte-derived DCs with contradictive results (51–53). Allavena et al. (51) demonstrated that IL-10 prevents DC differentiation by promoting a macrophage-like cell phenotype, whereas other studies reported that monocytes treated with IL-10 express markers associated with DCs (52, 53). Our group demonstrated that monocyte-derived DCs generated in the presence of IL-10 are a distinct subset of DCs with regulatory activities [(54), see next paragraph].

Interleukin-10 has been also applied to regulate already differentiated monocyte-derived immature (55) or matured (56, 57) DCs. In both settings, DCs exposed to IL-10 treatment express reduced levels of MHC class II and costimulatory molecules, show decreased Ag-presenting capacity, and become regulatory cells with the ability to promote anergic T cells (55, 56) with suppressive activity *in vitro* (57). More recently, it has been demonstrated that DCs matured in the presence of IL-10, termed IL-10-induced DCs, consist of two phenotypically and functionally distinct populations: CD83^high^CCR7^+^ and CD83^low^CCR7^−^ cells. The former cells display a strong migratory activity toward secondary lymphoid organs, have a stable phenotype, and induce *in vitro* T regulatory (Treg) cells with high suppressive activity. Based on these observations, the authors indicate that CD83^high^ CCR7^+^ IL-10-induced DCs are promising candidates for cell-based approaches to induce/restore tolerance *in vivo* (58).

## DC-10 A Distinct Population of Human Tolerogenic Dendritic Cells (tolDCs)

DC-10 are an inducible subset of human tolDCs characterized by the ability to secrete high levels of IL-10 in the absence of IL-12, and by the expression of a specific set of markers including CD14, CD16, CD11c, and CD11b, but not CD1a, M-DC8, or CD68 (54). Despite being generated from precursors in the presence of IL-10, DC-10 are mature cells expressing CD80, CD86, and HLA class II molecules. Importantly, DC-10 express a bunch of tolerogenic molecules such as ILT2, ILT3, ILT4, and HLA-G. Functional assays showed that, although DC-10 have a low stimulatory activity, they promote T cell anergy and induction of allo-specific T regulatory type 1 (Tr1) cells (50, 54, 59, 60). Tr1 cells are a subset of CD4^+^ T cells that co-express the integrin alpha2 subunit (CD49b) and the lymphocyte-activation gene 3 (LAG-3) (61), and secrete IL-10, TGF-β, variable amounts of IFN-γ and low/no IL-2, IL-4, and IL-17. Tr1 cells suppress immune responses *via* the secretion of IL-10, TGF-β, and of granzyme B [as reviewed in Ref. (13, 62)]. We demonstrated that DC-10 promote Tr1 cell differentiation *via* the IL-10-dependent ILT4/HLA-G pathway (54). Interestingly, DC-10-mediated induction of Tr1 cells is associated with high HLA-G expression (50).

DC-10 are present in peripheral blood and secondary lymphoid organs of healthy subjects and accumulate in human decidua in the first trimester of pregnancy (63). Interestingly, in peripheral blood of pregnant and non-pregnant women, the frequency of DC-10 is comparable, suggesting that either DC-10 migrate into decidua during pregnancy or are induced within the endometrium. Human decidua microenvironment is enriched in GM-CSF and IL-10 (64), both known to promote DC-10 differentiation, thereby decidual DC-10 can be either *de novo* induced from monocytes or derived from the conversion of resident decidual APCs. In the decidua of women with early miscarriage, DC-10 frequency is low (65), suggesting that in an inflammatory microenvironment differentiation of DC-10 is impaired. In line with this conclusion, in women with preeclampsia a subset of decidual CD14^+^DC-SIGN^+^ APCs with reduced HLA-G and ILT4 expression and impaired ability to promote Tregs *in vitro* have been identified. The authors speculated that the reduced IL-10 levels observed in preeclampsia may lead to reduced HLA-G and ILT4 expression and impaired tolerogenic activity of these CD14^+^DC-SIGN^+^ APCs (66).

An altered frequency of DC-10 has been reported in peripheral blood of cancer patients. In patients affected by acute myeloid leukemia, a significantly higher frequency of DC-10 compared with that observed in healthy donors was described. Interestingly, the percentage of DC-10 is higher in patients with HLA-G-expressing blasts compared with patients with HLA-G negative blasts (67). Even though the primary source of HLA-G was unclear, it was postulated that the presence of HLA-G-expressing DC-10 is involved in sustaining the expression of HLA-G on blasts contributing to inhibition of the immune system promoting tumor immune-escape. According to this hypothesis, an increased frequency of DC-10 expressing high levels of HLA-G has been identified in peripheral blood of patients with gastric cancer. Interestingly, the percentage of HLA-G^+^DC-10 strongly associates with advanced disease stage (68).

Overall, these studies indicate that DC-10 represent a subset of regulatory DCs contributing to IL-10-mediating tolerance and immune-escape.

## DC-10 As Inducers of Ag-Specific Tr1 Cells

**Figure 2:**
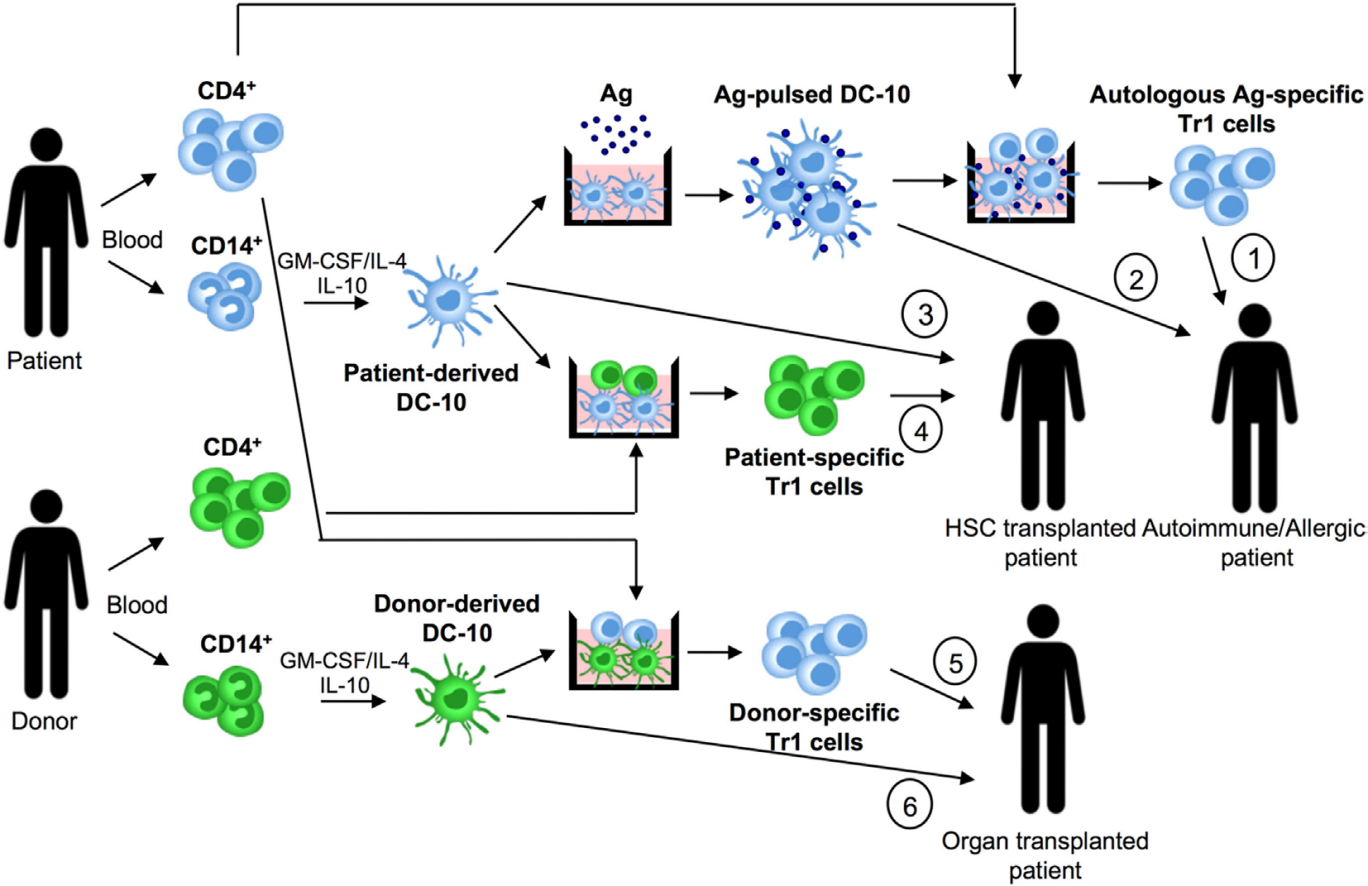
DC-10 and cell therapy approaches. DC-10 are differentiated *in vitro* from CD14^+^ cells in the presence of GM-CSF/IL-4/IL-10. In allergy and autoimmunity, patient-derived DC-10 pulsed with the specific antigen (Ag) can be used to induce differentiation of autologous Ag-specific T regulatory type 1 (Tr1) cell products (1), or directly infused into patients (2). In hematopoietic stem cell (HSC) transplantation, patient-derived DC-10 can be used to differentiate patient-specific Tr1 cell products (3), or directly infused into transplanted patients (4). In solid organ transplantation, donor-derived DC-10 can be used to promote differentiation of donor-specific Tr1 cell products (5), or directly infused into transplanted patients (6).

Dc-10 have entered the clinical arena as inducers of Ag-specific Tr1 cells for tailoring Treg-based cell therapy. We established and validated in Good Manufacturing Practice (GMP) conditions an efficient and reproducible *in vitro* method to generate, with minimal cell manipulation, allo-specific Tr1 cells (69, 70). Indeed, stimulation of T cells with allo-DC-10 induces a population of allo-specific Tr1 cells actively suppressing allo-specific effector T cells (50, 54, 59, 60). Recently, two improved GMP-compatible protocols using DC-10 have been developed for generating Tr1 cells for cell-based therapy. The first method generates allo-specific Tr1 cells (named T-allo10 cells, Bacchetta and Roncarolo, ClinicalTrials.gov identifier: NCT03198234) by culturing purified CD4^+^ T cells isolated from hematopoietic stem cell donor with patient-derived DC-10 in the presence of IL-10 (Figure 2). T-allo10 cells will be used as Tr1-based cell therapy in leukemia pediatric patients to prevent graft-versus-host disease (GvHD) (ClinicalTrials.gov identifier: NCT03198234). In the second protocol, CD4^+^ T cells isolated from patients on dialysis are cultured with donor-derived DC-10 in the presence of IL-10 to generate donor-specific Tr1-enriched cell medicinal product (named T10 cells) (Figure 2). T10 medicinal products will be injected in kidney transplant recipients to prevent graft rejection (60).

Stimulation of Th2 cells isolated from house dust mite allergic patients with autologous *in vitro* differentiated DC-10 pulsed with the allergen promotes their conversion of into IL-10-producing T cells (59). Moreover, DC-10 differentiated from monocytes of healthy subjects and peanut allergic patients and pulsed with relevant allergen induced the differentiation of peanut-specific Tr1 cells (71).

These findings indicate that patient-derived DC-10 can be *in vitro* pulsed with a given Ag and used to generate Ag-specific Tr1 cells for Treg-based cell approaches aim at restoring tolerance in allergy and autoimmune diseases.

## DC-10-Based Cell Therapy

The prominent role of DCs in promoting T-cell tolerance and the development of a GMP-compatible method to generate tolDC products allow their clinical application. Thus far, the few clinical trials performed demonstrated the safety and feasibility of tolDC-based cell therapies in the settings of autoimmunity and transplantation (72, 73). Nevertheless, the stability of the infused tolDC products and the maintenance of their tolerogenic properties *in vivo* remain open issues to be tackled for improving the safety and the efficacy of these therapies. Moreover, due to the increasing number of tolDCs that have been described, the optimal subset to be used as medicinal product is still to be defined. A comparative analysis of different populations of *in vitro* differentiated tolDCs examining their stability, cytokine production profile, and suppressive activity indicated that IL-10-modulated mature DCs are the best-suited cells for tolDC-based therapies (74, 75).

The observation that DC-10 are functionally more efficient than IL-10-modulated mature DCs in inducing hypo-responsiveness in allo-specific T cells (59) suggests that DC-10 represent a good alternative for cell-based approaches. Moreover, DC-10 are stable, since upon LPS stimulation, they maintain unaltered transcription profile and phenotype, and importantly the ability to induce Tr1 cells (76). DC-10 stability has been confirmed also *in vivo*, as their adoptive transfer modulates human T cell responses in a humanized mouse model. More recently, we demonstrated that DC-10 modulate allo iNKT cell induction and functions (Wu, under revision), indicating a broaden immunoregulatory function of DC-10, not limited to the CD4^+^ T cell compartment. The potency, stability, and widespread immunoregulatory activity of DC-10 make feasible their application in clinical setting. Specifically, autologous DC-10 pulsed with a given Ag and allo-DC-10 can be infused in patients to restore tolerance in autoimmune diseases and allergy and to prevent allograft rejection and GvHD, respectively (Figure 2).

## Conclusion and Perspectives

The discovery that DC-10 can be generated *in vitro* and induce Ag-specific Tr1 cell differentiation prompt their development as a tool for clinical approaches aimed at promoting/restoring Ag-specific tolerance in immune-mediated diseases. Protocols to generate alloAg-specific Tr1 cells with DC-10 for adoptive Tr1-based cell therapy have been developed and validated in GMP and are currently using in clinical applications. We believe that DC-10 represent a good candidate for DC-based therapies as they modulate effector immune responses, including pathogenic T cells, while leading to long-term tolerance *via* the *in vivo* induction of Ag-specific Tr1 cells. Studies in humanized mouse models are ongoing to establish the best route and dose of administration, lifespan, and homing kinetic of DC-10 and will be instrumental to design clinical protocols to test the safety and efficacy of DC-10-based cell therapy.

## Author Contributions

MC and GA wrote the manuscript. SG designed, supervised the drawing of the manuscript, and wrote the manuscript.

## Conflict of Interest Statement

The authors declare that the research was conducted in the absence of any commercial or financial relationships that could be construed as a potential conflict of interest.
